# Dissimilarity of Species and Forms of Planktonic *Neocalanus* Copepods Using Mitochondrial *COI*, *12S*, Nuclear *ITS*, and *28S* Gene Sequences

**DOI:** 10.1371/journal.pone.0010278

**Published:** 2010-04-28

**Authors:** Ryuji J. Machida, Atsushi Tsuda

**Affiliations:** Ocean Research Institute, University of Tokyo, Tokyo, Japan; University of Sydney, Australia

## Abstract

**Background:**

A total of six *Neocalanus* species inhabit the oceans of the world. Of these, three species plus form variants (*N. cristatus, N. plumchrus, N. flemingeri* large form, and *N. flemingeri* small form), which constitute a monophyletic group among *Neocalanus* copepods, occur in the Northwestern Pacific off Japan. In the present study, we have tried to discriminate the three species plus form variants of *Neocalanus* copepods based on sequences of four DNA marker regions.

**Methodology/Principal Findings:**

Discrimination was performed based on the DNA sequence information from four genetic markers, including the mitochondrial *COI*, *12S*, nuclear *ITS*, and *28S* gene regions. Sequence dissimilarity was compared using both distance- and character-based approaches. As a result, all three species were confirmed to be distinct based on the four genetic marker regions. On the contrary, distinction of the form variants was only confirmed based on DNA sequence of the mitochondrial *COI* gene region.

**Conclusions/Significance:**

Although discrimination was not successful for the form variants based on the mitochondrial *12S*, nuclear *ITS*, and *28S* genes, diagnostic nucleotide sequence characters were observed in their mitochondrial *COI* gene sequences. Therefore, these form variants are considered to be an important unit of evolution below the species level, and constitute a part of the *Neocalanus* biodiversity.

## Introduction

There is now a general consensus that variations in DNA sequences contain useful information for taxonomic studies [1]–[4]. DNA barcoding has attempted to lead the establishment of DNA sequence-based species identification technology (http://www.barcoding.si.edu/). Although expectations of DNA barcoding technology are high [5]–[7], careful discussion of several aspects is still needed to make the technique fool-proof. In this paper, we focus on two subjects that need to be clarified to use DNA sequence information for DNA barcoding, 1) the species concepts, and 2) technical difficulties of genetic analysis. In addition to these two issues, one of the important considerations is how to compare the DNA sequences and to decide if the compared sequences are derived from the same or different species, in other words, application of distance- or character-based identification [8]. In this context, we compiled DNA sequence data, using *Neocalanus* calanoid copepods, using not only a distance-based but also a character-based approach.

The copepod genus *Neocalanus* includes six species and one form in the world oceans [9], [10], [11]. Of the six species, three species (*N. cristatus, N. plumchrus,* and *N. flemingeri*), which form a monophyletic group, are known to occur in the subarctic Pacific [12]. All of the *Neocalanus* species that inhabit the subarctic Pacific are known to perform extensive ontogenetic vertical migrations, occurring and growing in the euphotic layer from winter to summer and descending to the meso- and bathypelagic layers from summer to winter (or autumn) for maturation and spawning [11], [13]–[18].

*Neocalanus flemingeri* was not described until the year 1988 [10] because of the morphological resemblance to its sibling species, *N. plumchrus*. About a decade later, the existence of large-bodied individuals within *N. flemingeri* was reported from samples collected in the Northwestern Pacific off Japan and in the Okhotsk Sea [11], [16]. Tsuda et al. [18] further reported that the large-form individuals, occurring in the Northwestern Pacific off Japan, are advected from the Okhotsk Sea. As a consequence, a hierarchical assemblage of forms, sibling species, and species, occur in a single locality off northeast Japan in the Northwestern Pacific. In the present study, genetic distances and characteristics between the forms (*N. flemingeri* large and small forms), sibling species (*N. flemingeri* and *N. plumchrus*), and species (*N. cristatus* and *N. flemigeri, N. plumchrus*) were estimated using four genetic markers (mitochondrial *COI*, *12S*, nuclear *ITS*, and *28S*) and discrimination of the taxonomic hierarchies was performed.

## Results

### Genetic dissimilarity between the forms, sibling species, and species of *Neocalanus*

**Figure 1:**
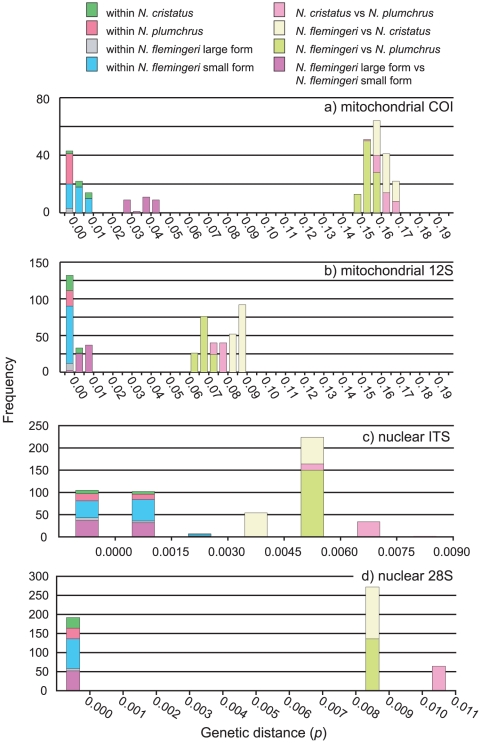
Mismatch distribution of pairwise genetic distances (*p*) of the three *Neocalanus* species, including two forms. Bar colours indicate the compared genetic distances between and within each species or form.

Genetic distances of the four marker regions, mitochondrial *COI*, *12S*, nuclear *ITS*, and *28S* were estimated for comparisons among the forms of *N. flemingeri*, sibling species (*N. flemingeri* and *N. plumchrus*), and species (*N. cristatus, N. plumchrus,* and *N. flemingeri*) (Table 1). Mean genetic distances (*p*) of the mitochondrial *COI* region between the forms (*N. flemingeri* large and small forms) and sibling species (*N. plumchrus* and *N. flemingeri*), were 0.036 and 0.154, respectively. The largest genetic distance (*p*) was observed in the comparisons between species (*N. cristatus* vs *N. flemingeri* and *N. cristatus* vs *N. plumchrus*; 0.162 and 0.162, respectively). Mean genetic distances within forms and species were all smaller than 0.010 (Fig. 1). In contrast to the mitochondrial *COI* region, mean genetic distances of the mitochondrial *12S* region were relatively small for all taxonomic comparisons. Between the forms and sibling species, genetic distance comparisons for this region were 0.005 and 0.067 (Table 1). Values were 0.086 and 0.076 in the comparisons of *N. cristatus* vs *N. flemingeri* and *N. cristatus* vs *N. plumchrus*, respectively. Distances within forms and species were all smaller than 0.005 (Fig. 1). Compared to the mitochondrial genes, genetic distances for the nuclear regions were all small. Mean genetic distances (*p*) of the nuclear *ITS* region were 0.001 and 0.005 in comparisons of the forms and sibling species, respectively. Mean genetic distances between species were 0.004 and 0.007 in comparisons of *N. cristatus* vs *N. flemingeri* and *N. cristatus* vs *N. plumchrus*, respectively. Mean genetic distances for within species and forms were all smaller than 0.002 (Fig. 1). Mean genetic distances of the nuclear *28S* region were 0.000 and 0.008 in the comparison of the forms and sibling species, respectively (Table 1). Mean genetic distances for the species were 0.008 and 0.011 in comparisons of *N. cristatus* vs *N. flemingeri* and *N. cristatus* vs *N. plumchrus*, respectively. Within-population genetic distances were all 0.000.

**Table 1:** Mean genetic distances (*p*) between the species and forms of *Neocalanus*.

Comparison between	*COI* (SD)	*12S* (SD)	*ITS* (SD)	*28S* (SD)
*N. flemingeri* large VS *N. flemingeri* small	0.03586 (0.00541)	0.00451 (0.00209)	0.00058 (0.00063)	0.00000 (0.0000)
*N. flemingeri* VS *N. plumchrus*	0.15379 (0.00286)	0.06703 (0.00265)	0.00533 (0.00063)	0.00813 (0.00001)
*N. flemingeri* VS *N. cristatus*	0.16190 (0.00314)	0.08559 (0.00162)	0.00430 (0.00074)	0.00811 (0.00000)
*N. cristatus* VS *N. plumchrus*	0.16151 (0.00358)	0.07596 (0.00195)	0.00659 (0.00070)	0.01081 (0.00001)

Distances of four genetic marker regions were estimated for the mitochondrial *COI*, *12S*, nuclear *ITS* and *28S*.

**Figure 2:**
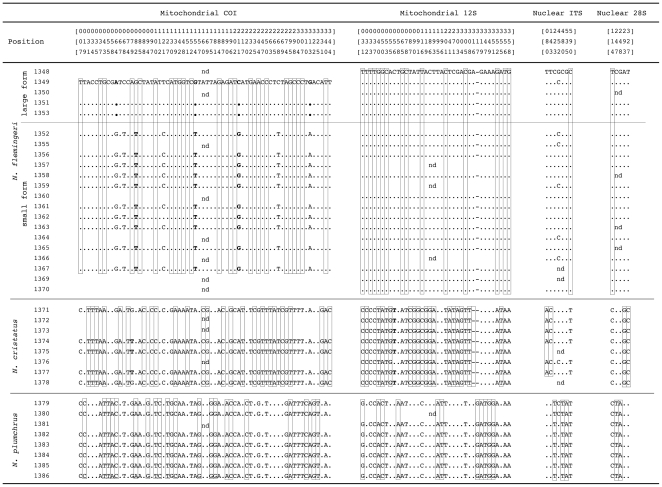
Aligned sequences of mitochondrial *COI*, *12S*, nuclear *ITS*, and *28S* regions of the *Neocalanus* copepods. Only sites in which variations were observed in two individuals or more were included. Open box sites are the DNA regions that are “purely” diagnostic to each species. Bold letters indicate the “private” sites to each species (terminology described in Sarkar et al., 2002) [32].

In addition to the analyses based on genetic distance, we have also compiled the observed sequence character variations (Fig. 2). In the figure, only those sites exhibiting variation in two individuals or more were included. Sites exhibiting multiple peaks in any individuals were deleted from the analysis. At the mitochondrial *COI* region, 64 sites showed variation in more than one individual. Of the 64 sites, only three sites (site 068, 121, and 268) did not contain any information to characterize either species or forms. In the mitochondrial *12S* region, 38 variable sites were observed and all sites had information that supported the species as distinct. In the nuclear regions, seven and five variable sites were observed in *ITS* and *28S*, respectively, and all had information to characterize the species.

## Discussion

### Species discrimination of the *Neocalanus* copepods based on DNA sequences of *COI*, *12S*, nuclear *ITS*, and *28S* regions

Although the biological species concept (BSC; groups of actually or potentially inter-breeding natural populations, which are reproductively isolated from other such groups) [19], is accepted by most zoologists, species descriptions and names are most often based on morphological difference that should be shown to be non-overlapping between species. If the mating of these species is sympatric and synchronic, we have good evidence that the morpho-species are good biological species that are reproductively isolated and not interbreeding. On the contrary, it is difficult to describe these morpho-species as “biological species” once their mating is allopatric or allochronic, because of requirement for performing the mating experiment, which is the true test of the primary biological species concept based on “reproductive isolation”. In the case of three *Neocalanus* species, life history and distribution data allow us to infer that these species are reproductively isolated biological species [10], [11], [13]–[17]. How can this taxonomic status be verified through DNA sequence information? Distance-based analyses reveal divergence gaps between inter- and intra-specific distances in the mitochondrial *COI*, *12S*, and nuclear *28S* regions (Fig. 1). In addition to the distance-based comparison, diagnostic sequence character variations were observed between species in all four regions (Fig. 2). The smallest number of unique characters was observed in the nuclear *28S* region, in which at least three character differences were observed between each species. From these results, the distinctness of species was also indicated by character-based comparisons in all four-marker regions. These results indicate that the genetic data further reinforced identification of the three *Neocalanus* species.

**Figure 3:**
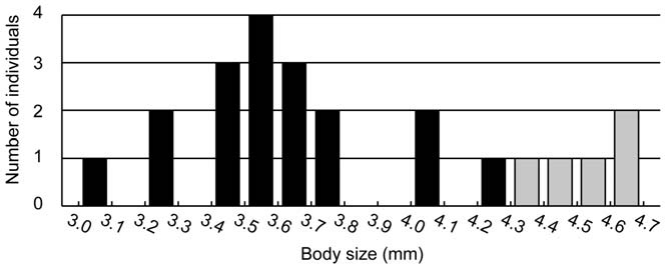
Prosome length frequency distribution for *Neocalanus flemingeri*. Individuals assigned to the small and large forms based on mitochondrial *COI* gene sequence are indicated by black and shaded bars, respectively.

In contrast to the species-level comparisons, the taxonomic status of the forms of *N. flemingeri* were not clarified by either morphological or genetic data. Between the forms of *N. flemingeri*, no diagnostic morphological character has been identified [11]. The only known morphological difference between the forms is body size. In the present study, body size between the two forms does not overlap but is not highly different (Fig. 3). It is probable that increasing sample size will find specimens that overlap in size between forms. Furthermore, it is not appropriate to use this quantitative morphological difference, which is unrelated to reproduction, for a diagnostic character for identification based on the BSC. Given this context, these forms of *N. flemingeri* were not clearly distinct as species based on morphological characters.

How can we clarify the taxonomic status of these forms based on genetic information? In addition to morphological characteristics, the ambiguous taxonomic status of these forms was also indicated by genetic data. In the distance-based comparisons, divergence gaps were observed between the intra- and inter-form genetic distances based on the mitochondrial *COI* gene sequences (Fig. 1). In contrast to the mitochondrial *COI* region, mitochondrial *12S*, nuclear *ITS*, and *28S* regions did not show clear differences in genetic distances between intra- and inter-form comparisons. As in the distance-based comparison, the character-based approach discriminates the forms only based on the mitochondrial *COI* gene sequences (Fig. 2). Can we answer the question as to whether or not these forms are distinct species, based on the BSC? Are they reproductively isolated or not? In the present study, we have collected both forms of *N. flemingeri* at a single locality, off the Sanriku area of Japan. However, the main distributions of the large- and small-form population are in the Okhotsk Sea and subarctic Pacific, respectively [18]. Therefore, mating in the main populations of these two forms takes place allopatrically. In this context, the extent of the reproductive isolation is unknown since a true test of the biological species concept, the mating experiment, which is very difficult to perform for these pelagic animals, is required to answer the question. This taxonomic ambiguity is a recurring problem under the BSC when populations are allopatric or allochronic, due to spatial or temporal separation of reproduction. Therefore, ambiguity of species based on the BSC always remains with increasing the sample coverage from various locations. Although we have failed to identify the taxonomic status of the form variants, it is an important unit of evolution, and constitutes part of the *Neocalanus* biodiversity.

### Practical difficulties of using each gene region as a species identification marker

Mitochondrial genes are considered good candidates for a species identification marker. However, the genome has three major drawbacks that potentially limit its use in this context. First is the existence of nuclear mitochondrial pseudogenes (numts). Numts are DNA sequences paralogous to mitochondrial DNA, and integrated into the nuclear genome [20]. The paralogous nature of numts can lead to incorrect identification, when sequences of numts are determined accidentally. Second is the occurrence of mitochondrial introgression. After the occurrence of hybridization, lineage sorting of the mitochondrial genome is much more efficient than nuclear markers due to the difference in effective population sizes of these two genomes [21]. Between species that have experienced mitochondrial introgression, mitochondrial gene sequence data will have fewer diagnostic characters for each species. Third is the pattern of descent, via maternal inheritance. In general, the mitochondrial genome is inherited only maternally. In most cases, dispersal of a single species does not show a male or female bias. Therefore, clustering based on the mitochondrial gene marker represents the population structure of effective populations. However, if males have a higher dispersal ability than females, a maternally-inherited mitochondrial marker might represent false clustering, and such a sex-biased dispersal has been reported in several animals [e.g. 22]–[24]. As a result, in theory, the discrimination of species based solely on mitochondrial genetic marker information has shortcomings, since it holds only maternal information.

Nuclear genome regions also have some drawbacks as species identification markers. The nuclear markers adopted in the present study, *ITS* and *28S* regions, have multiple copies in the nuclear genome. If concerted evolution is incomplete, they will be polymorphic. In such individuals, clear nucleotide sequences are not possible to determine without cloning the PCR products.

In the present study, we sequenced not only the mitochondrial *COI* and *12S*, but also the nuclear *ITS*, and *28S* regions. From the present results, identification of species was feasible based on the sequences of all four marker regions. Taking into account the practical difficulties discussed above, there are two reasons why it is advisable to use multiple genome information for species identification. The first reason is the theoretical context, as discussed above, that the mitochondrial genome only holds information from maternal descent. The second reason is to avoid practical difficulties in obtaining useful data, such as numts, mitochondrial introgression, and incomplete concerted evolution. By analyzing two genomes, misidentification caused by these difficulties can be verified.

### Population subdivision observed for the forms of *N. flemingeri*

Genetic divergence between *N. flemingeri* form variants was observed in the mitochondrial *COI* gene sequences. Diagnostic sequence characters were also observed in the mitochondrial *12S* region between the two forms, but these were deleted from figure 1 because of the occurrence of ambiguous sites in some individuals.

Much discussion has been carried out in the literature about the status of the large and small forms of *N. flemingeri*. Kobari and Ikeda [16] discussed sexual dimorphism as a probable reason for the length polymorphism observed for C4 and C5 individuals. On the other hand, Tsuda et al. [11] pointed out the possibility of two genetically diverged populations, which correspond to the length polymorphisms. Based on the results of the present study, it has become clear that these length polymorphisms are genetically distinct populations.

As discussed above, we have collected both forms of *N. flemingeri* at a single locality, off the Sanriku area of Japan. However, the distribution of the main population of large-form individuals is restricted to the Okhotsk Sea [11], [18]. Most probably, large-form individuals collected in the present study were advected from the Okhotsk Sea. Norris [25] summarized five speciation models for pelagic animals, including allopatric, parapatric, vicariance, depth parapatric, and seasonal sympatric speciation. Based on the present results, the mechanism of the form subdivision is still an open questions. However, depth parapatry and seasonal sympatry might play important roles in the population subdivision, both of which involve speciation without the impenetrable barrier to gene flow of the vicariance speciation model. *N. flemingeri* inhabits the subarctic Pacific and its marginal seas. All of the seas have unique hydrographic characteristics that vary seasonally, which might produce independent populations with life cycles adapted to each sea.

Machida et al. [12] pointed out the difference in the number of species of *Neocalanus* copepods present in the subarctic Pacific (three) and Subantarctic (one), and discussed the importance of marginal seas in establishing new populations. Based on the results of the present study, the importance of marginal seas for species diversification is further emphasized. The next step in inferring the mechanism of pelagic speciation is to identify the geographic location of the populations having the most ancestral clade among the large-form individuals. The location is the most probable site where population subdivision occurred, and observation of mating behavior in the location would give clues to the diversification mechanism occurring in those marginal seas.

## Materials and Methods

### Animal samples and DNA extraction

All specimens used for the analysis were collected from 1033 meters to the surface in the Northwestern Pacific off the Sanriku area (40°01.2′ N 145°01.5′ E) with a plankton net equipped with a 2 m^2^-mouth opening and 0.69-mm mesh (July 3, 2006). Specimens were preserved in 99.5% ethanol immediately after collection. After identification, total genomic DNA was extracted from the first antenna using the Gentra Puregene Tissue Kit (Qiagen). A total of 37 individuals including eight individuals of *N. cristatus*, eight individuals of *N. plumchrus*, sixteen individuals of *N. flemingeri* small form, and five individuals of *N. flemingeri* large form were analyzed in this study.

### PCR and sequencing

Mitochondrial *COI*, *12S*, nuclear *ITS*, and *28S* sequences were amplified using polymerase chain reaction (PCR). Since precise positioning of the nuclear *ITS* region was not determined from transcript analysis of rRNAs, not only the *ITS1* and *ITS2* but also 5.8S rDNA, and a portion of 28S rDNA are included in the so called *ITS* region in the present study. Four sets of primers were used for the present study [(mitochondrial *COI*; LCO1490, HCO2198 [26]) (mitochondrial *12S*; H13842-12S, L13337-12S [27], [28]) (nuclear *ITS*; 1368-18S, R329-28S [29]) (nuclear *28S*; F352-28S, R768-28S)]. Three primers, R329-28S (5′-acg gta ctt gtt kac tat cgg tct-3′), F352-28S (5′-aga ccg ata gtm aac aag tac cgt-3′), and R768-28S (5′-tag act cct tsg tcc gtg ttt ca-3′) were newly designed based on aligned sequences from *Homo sapiens* (NR_003287), *Drosophila melanogaster* (NR_004369), *Tigriopus californicus* (AF363324), and *Mus musculus* (BK000964).

PCR was done in a Model 9700 thermal cycler (Applied Biosystems) and reactions were carried out with 40 cycles of a 15-µl reaction volume containing 9.95 µl of sterile, distilled H_2_O, 1.50 µl of 10X buffer, 1.20 µl of dNTP (2.5 mM each), 0.60 µl of each primer (5 µM), 0.15 µl of Z Taq (Takara), and 1.00 µl of template. Two types of thermal cycle profiles were applied. First, the thermal cycle profile applied for mitochondrial *COI* and *12S* was that of a “standard three step PCR”: denaturation at 94°C for 10 s, annealing at 45°C for 10 s, and extension at 72°C for 10 s. The second thermal cycle profile, applied for nuclear *ITS* and *28S*, was that of “shuttle and touchdown PCR”; denaturation at 94°C for 5 s, and annealing and extension combined at the same temperature (70°C) for 20 s. Temperature for the annealing and extension were progressively decreased with advancing cycles (−0.5°C per cycle) from 70 to 65°C during the first 10 cycles and the kept constant at 65°C during the subsequent 30 cycles. PCR products were electrophoresed on a 1.0% L 03 agarose gel (Takara) and later stained with ethidium bromide for band characterization using ultraviolet transillumination. PCR products were purified with ExoSap-IT (GE Health BioScience), following the manufacturer's protocol, and were subsequently used for direct cycle sequencing with dye-labeled terminators (Applied Biosystems). All sequencing reactions were performed according to the manufacturer's instructions. Labeled fragments were analyzed on a model 3130XL sequencer (Applied Biosystems).

### Sequence analysis

DNA sequences were analyzed using the computer software program Geneious version 4.5.5 (Biomatters Ltd.). MacClade 4.0 was used in various phases of the analysis [30]. During the course of sequence analysis, we occasionally observed clear double peaks in electropherograms, which might be the effect of nuclear mitochondrial pseudogenes (numts). Such ambiguous peaks were coded in IUPAC codes. We relied on the diagnostic value of Geneious for the ambiguous electropherogram analysis and did not make any corrections. Genetic distances (*p*) were estimated using PAUP 4.0b10 [31]. For calculation of genetic distances, ambiguous data were ignored for affected comparisons. We have also deleted sites from the sequence alignment in figure 1, if any individuals in the present study showed double peaks in the position. Diagnostic sequence characters were also observed in the mitochondrial *12S* region between the two forms, but were deleted from figure 1 due to the occurrence of ambiguous sites in some individuals. All sequences determined during the present study were deposited in the databases of the International Nucleotide Sequence Collaboration (accession number AB526881-AB527004).
